# Isolation and Biophysical Characterization of Lipoxygenase-1 from Soybean Seed, a Versatile Biocatalyst for Industrial Applications

**DOI:** 10.3390/biom16010162

**Published:** 2026-01-19

**Authors:** Ioanna Gerogianni, Antiopi Vardaxi, Ilias Matis, Maria Karayianni, Maria Zoumpanioti, Thomas Mavromoustakos, Stergios Pispas, Evangelia D. Chrysina

**Affiliations:** 1 Institute of Chemical Biology, National Hellenic Research Foundation, 48 Vassileos Constantinou Ave., 11635 Athens, Greece; igerogia@eie.gr (I.G.); iliasmatis@eie.gr (I.M.); mariaz@eie.gr (M.Z.); 2 Department of Chemistry, National and Kapodistrian University of Athens, Panepistimioupolis Zografou, 15772 Athens, Greece; tmavrom@chem.uoa.gr; 3 Theoretical and Physical Chemistry Institute, National Hellenic Research Foundation, 48 Vassileos Constantinou Ave., 11635 Athens, Greece; avardaxi@eie.gr (A.V.); mkaragia@eie.gr (M.K.)

**Keywords:** soybean lipoxygenase-1, biocatalysts, protein purification, biophysical characterization, physicochemical properties

## Abstract

Lipoxygenases are enzymes found in plants, mammals, and other organisms that catalyse the hydroperoxidation of polyunsaturated fatty acids, such as arachidonic, linoleic, and linolenic acids. They have attracted a lot of attention as molecular targets for industrial and biomedical applications, due to their implication in key biological processes, such as plant development and defence, cell growth, as well as immune response and inflammation. Soybean (*Glycine max*) lipoxygenase (LOX) is a versatile biocatalyst used in biotechnology, pharmaceutical, and food industries. sLOX1, a soybean LOX isoform, is central in various industrial applications; thus, it is of particular interest to develop an efficient sLOX1 isolation process, control its activity, and leverage its potential as an effective industrial biocatalyst, tailoring it to a specific desired outcome. In this study, sLOX1 was extracted and purified from soybean seeds using an optimized protocol that yielded an enzyme preparation with higher activity compared to the commercially available lipoxygenase. Comprehensive biophysical characterization employing dynamic and electrophoretic light scattering, fluorescence, and Fourier-transform infrared spectroscopies revealed that sLOX1 exhibits remarkable structural and functional stability, particularly in sodium borate buffer (pH 9), where it retains activity and integrity up to at least 55 °C and displays minimal aggregation under thermal, ionic, and temporal stress. In contrast, sLOX1 in sodium phosphate buffer (pH 6.8) remained relatively stable against ionic strength and time but showed thermally induced aggregation above 55 °C, while in sodium acetate buffer (pH 4.6), the enzyme exhibited a pronounced aggregation tendency under all tested conditions. Overall, this study provides physicochemical and stability assessments of sLOX1. The combination of enhanced catalytic activity, high purity, and well-defined stability profile across diverse buffer systems highlights sLOX1 as a promising and adaptable biocatalyst for industrial applications, offering valuable insights into optimizing lipoxygenase-based bioprocesses.

## 1. Introduction

Lipoxygenases (LOXs) are enzymes that exist in different organisms such as mammals and plants [1,2,3]. Mammalian LOXs are mainly involved in cell development and proliferation, carcinogenesis, inflammation, cardio-vascular and central system dysfunction, and metabolic disorders such as diabetes [4,5,6,7]. In plants, LOXs play a central role in mechanical wounding, function as storage proteins, while previous studies have also shown that they serve in a protective role when overexpressed in response to pathogens and insects’ attack [2,8,9]. LOXs are also widely used biocatalysts in industrial processes, particularly in the food industry, where their oxidative activity plays a central role in product quality and functionality [10]. For instance, LOXs contribute to both beneficial and detrimental effects—such as the co-oxidation of carotenoids like *β*-carotene leading to nutrient loss, the oxidative and hydrolytic deterioration of lipids, and the formation of desirable or off-flavours in soybean-based foods [11,12,13,14,15].

LOXs belong to the superfamily of dioxygenases with non-heme iron, which is either in the inactive Fe^+2^ oxidation state or at the active Fe^+3^ one. They catalyse the regio- and stereo-specific peroxidation of polyunsaturated fatty acids with a *cis,cis*-1,4-pentadiene moiety, such as arachidonic acids, linoleic acid (LA), and linolenic acid. This reaction leads to the formation of unsaturated fatty acid hydroperoxides such as hydroxy-eicosatetraenoic acids (HETEs) and leukotrienes (LTs) [16,17,18]. There are many different isoforms of lipoxygenases, which are named after either the position of the peroxidation and stereospecificity of the product, in the case of mammals, or the enzymatic characteristics and structural features in the case of plants [3]. Due to LOXs’ abundance in plants, soybean LOXs have been investigated to improve understanding of their structure-function relationships and shed light on their inhibition by natural antioxidant compounds, producing new knowledge that would be useful for other organisms, including humans [13]. Previous studies showed that there are four main isoenzymes in soybean seeds, LOX1, 2, 3a, and 3b. LOX3a and 3b have similar behaviour and composition; thus, they are named LOX3 and are considered as a single type. Soybean LOX1 (sLOX1) comprises 834 residues (MW 94 kDa), sLOX-2 has 859 (MW 96.5 kDa), and sLOX-3 has 865 (MW 97 kDa) [19]. LOX1 and LOX3 share a very high structural homology with a root mean square deviation (r.m.s.d.) of 1.2 Å calculated by the Dali server [20,21,22]. Despite the high sequence similarity among isoforms, the subtle structural differences observed in the accessibility of the cavities adjacent to the iron binding site may account for their specificity/preference of substrate [23,24]. sLOX1 was found to have optimum activity at pH 9.0, while sLOX2 and sLOX3 have optimum activity at slightly acidic conditions, at pH ranging from 6 to 7 [25]. In the present study, the isoform of sLOX1 was isolated and purified from soybean seed extract with the aim of studying its biophysical properties.

Previous literature on soybean protein isolate (SPI) focused on biophysical studies that aimed to uncover the mechanism underlying LOXs’ (including sLOX1) implication in the oxidation of other proteins present in SPI. This oxidation was facilitated by the presence of lipid residues like LA and affects the quality of soybean-based food. More specifically, the influence of LOX and LA in the SPIs was investigated through the analyses of their biophysical properties, such as surface hydrophobicity, intrinsic fluorescence, size, structure, and aggregation through Dynamic Light Scattering (DLS) and electron microscopy [26,27]. The catalytic site and the oxidation state of iron have been investigated with Electron Paramagnetic Resonance (EPR) and Circular Dichroism (CD) spectroscopies, by studying a wild-type recombinant sLOX1, and mutated variants with iron-interacting-residues alternations [28,29]. Recombinant sLOX was also studied using isothermal titration calorimetry (ITC), DLS, and Differential Scanning Calorimetry (DSC) to investigate the influence of oleyl sulfate, a fatty acid derivative, on the structure and activation of soybean LOX [30]. In this study, sLOX1 was purified from soybean seeds employing a modified protocol of previously reported studies [25,31,32]. The refined extraction and purification procedure significantly improved process efficiency, reducing the preparation time by 50% and enhancing the enzyme yield by approximately 60%. The biophysical properties of the purified enzyme were examined in the presence of three different buffer solutions: sodium acetate buffer, pH 4.6, sodium phosphate buffer, pH 6.8, and sodium borate buffer, pH 9.0, selected based on their prior use in biochemical and structural studies of the enzyme, with the ionic strength, pH and temperature being the major variables, using Dynamic and Electrophoretic Light Scattering (ELS) (*ζ* potential). The structural properties of sLOX1 were also investigated using fluorescence and Fourier Transform Infrared (FTIR) spectroscopies. Furthermore, the effects of temperature and ionic strength on the structural stability and aggregation behaviour of the enzyme solutions were investigated, along with their time-dependent storage stability, to improve our understanding of the enzyme’s physicochemical properties.

## 2. Materials and Methods

### 2.1. sLOX1 Extraction, Pretreatment, and Purification

**Figure 1:**
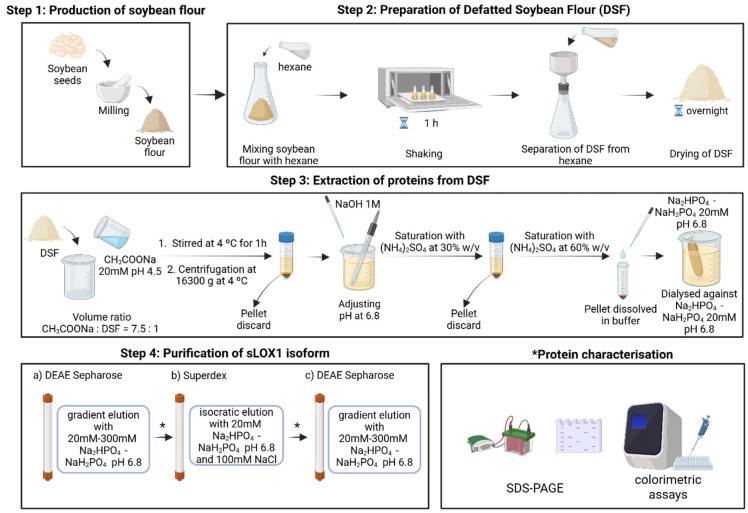
Schematic representation of the extraction and purification steps followed for the preparation of sLOX1, created in BioRender. Chrysina, E. (https://BioRender.com/qzm2gj8 (accessed on 29 December 2025)) is licensed under CC BY 4.0. Protein characterisation was performed after each purification stage as indicated with a (*) in Step 4.

sLOX1 was extracted from organic soybean seeds using a modified protocol that was previously established [25,31,32]. Soybean seeds were soaked in drinking water for 24 h. The hydrated seeds were peeled and left to dry before pulverisation. The soy flour was then defatted by mixing and shaking with 100% *v*/*v* hexane at a ratio of 1:3 *w*/*v* flour-to-solvent for 1 h, at 25 °C. The suspension was subjected to filtering using a Buchner funnel and was left to dry in the open air. The defatted soybean flour was treated as described by Axelrod et al. [25], and the mixture of extracted proteins was received at a 20 mM sodium phosphate buffer, pH 6.8. Isolation of sLOX1 from the mixture of the extracted proteins was performed by anion exchange chromatography as previously described by Axelrod and coworkers [25]. For this first purification step, a DEAE Sepharose column (5 mL) was equilibrated with 20 mM sodium phosphate buffer, pH 6.8. The total volume of extracted protein solution loaded on the column was 45 mL in 20 mM sodium phosphate buffer, pH 6.8. An elution gradient was created by combining different concentrations of sodium phosphate buffer, pH 6.8 (i.e., 50 mL of 20 mM with 50 mL of 300 mM). Flow rate was set at 24 mL/h for ~4.5 h. Eluted fraction volume was 1.5 mL. Fractions enriched in sLOX1 were identified via a colourimetric assay selective for sLOX1 (described in detail in Section 2.2) and 14% *w*/*v* SDS-PAGE gel. sLOX1-containing fractions were pooled and concentrated to a final volume of 0.5 mL and loaded into a HighLoad Superdex200 16/60 column (Cytiva, Marlborough, MA, USA) for size exclusion chromatography (SEC). The column was previously equilibrated with a 20 mM sodium phosphate, pH 6.8 buffer and 100 mM sodium chloride solution. For the isocratic elution, 120 mL of the same buffer was used, at a flow rate of 30 mL/h for ~4 h. The volume of each collected fraction was 2.5 mL. Fractions from the peak showing the highest absorbance value at 280 nm (Abs_280_) and high activity via colourimetric assay were pooled and dialysed overnight against 20 mM sodium phosphate buffer, pH 6.8. Following dialysis, anion exchange chromatography was again used, as the final purification step, using the same running conditions as in purification step 1, though with a flow rate of 15 mL/h and an eluted fraction volume of 1 mL each. The elution gradient ran for approximately 7 h, and the fractions containing sLOX1 were pooled and concentrated to a final concentration of 2.5 mg/mL in 20 mM sodium phosphate buffer, pH 6.8. The steps followed from the production of soybean flour to the protein characterisation are summarised in Figure 1.

### 2.2. Colourimetric Detection Assays

**Figure 2:**
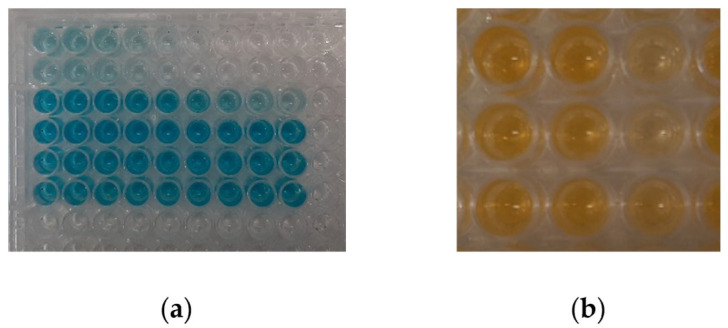
Visual inspection of (**a**) methylene blue bleaching observed from the colourimetric detection assay for isoforms sLOX1 and sLOX2 at a 96-well plate and (**b**) *β*-carotene bleaching as a positive result of the colourimetric detection assay for sLOX3 isoform.

The selective detection of sLOX1 throughout the extraction, pretreatment, and purification process was performed using an automated spectrophotometric method, initially described by Suda and coworkers [33], which associated the existence of the distinct sLOX isoforms with the bleaching activity on methylene blue (Figure 2a). The reaction was initiated by the addition of 0.05 mL protein sample to a mixture containing 100 mM sodium borate buffer (pH 9.0), 0.01 mM methylene blue, 1 mM sodium linoleate buffer, which is the substrate of the reaction, and distilled water to a final total volume of 1 mL. The reaction was monitored for 10 min at 25 °C by transferring 200 μL of the reaction mixture at time intervals of 15 s, 1 min and 10 min to a 96-well plate, containing 50 μL of SDS 10% *w*/*v* in each well and measuring the absorbance at 660 nm using a Varioskan Flash plate reader by Thermo Fisher Scientific, Waltham, MA, USA, installed at the Instruct-EL hub, at the National Hellenic Research Foundation (NHRF,). The implementation of appropriate equipment allows the automated measurement recording and the reduction in the required sample volume to 50 μL per measurement. The observed decrease in the absorbance was correlated with the progress of the hydroperoxidation of linoleic acid, which indicated the presence of sLOX1, which exhibits optimum activity at pH 9.0. For comparative purposes, the activity of the purified sLOX1 was assessed alongside a commercial soybean lipoxygenase standard (Type I-B, ≥50,000 units/mg solid; Sigma-Aldrich, St. Louis, MO, USA, CAS 9029-60-1) under identical assay conditions. Absorbance changes were monitored spectrophotometrically, and enzyme activity was expressed in terms of ΔAbs per minute.

The presence of isoform sLOX2 was also investigated using a previously established protocol by Suda and coworkers [33]. The reaction was initiated by the addition of 0.05 mL protein sample to a mixture of 80 mM sodium phosphate buffer (pH 6.8), 0.01 mM methylene blue, 20 mM DTT, 10% *v*/*v* acetone, 1 mM sodium linoleate buffer, and distilled water to a final volume of 1 mL. The reaction was monitored for 15 min at 25 °C by transferring 200 μL of the reaction mixture at time intervals of 15 s, 1 min and 10 min to a 96-well plate, containing 50 μL of SDS 10% *w*/*v* in each well and measuring the absorbance at 660 nm using a Varioskan Flash plate reader by Thermo Fisher Scientific like in the case of sLOX1. The decrease in the absorbance at 660 nm over time indicated the progress of the reaction, which was associated with the presence of sLOX2, which exhibits optimum activity at pH 6.8.

For the detection of isoform sLOX3, according to Suda and coworkers’ protocol [33], 0.05 mL protein sample was added to a mixture of 100 mM sodium phosphate buffer (pH 6.6), 10% *v*/*v β*-carotene, 1 mM sodium linoleate buffer, and distilled water to a final volume equal to 1 mL. The reaction was monitored for 15 min at 25 °C by transferring 200 μL of the reaction mixture at time intervals of 15 s, 1 min, and 10 min to a 96-well plate, containing 50 μL of SDS 10% *w*/*v* in each well and measuring the absorbance at 452 nm with the same plate reader. The decrease in the absorbance at 452 nm over time indicated the progress of the reaction, which was associated with the presence of sLOX3 (Figure 2b).

### 2.3. Biophysical Characterisation

The biophysical properties of sLOX1 were studied at three different buffers: sodium acetate 0.2 M pH 4.6, sodium phosphate 0.02 M pH 6.8, and sodium borate 0.2 M pH 9.0. For the buffer exchange, Satorius Vivaspin Turbo 4, Göttingen, Germany centrifugal concentrators with a 10 kDa MW cutoff were used. All the solutions were filtered using hydrophilic PVDF filters with 0.25 μm mesh to remove any dust particles or large aggregates, before measurement. Graphical representation of the results was performed with the software OriginLab (Version 2021b, OriginLab Corporation, Northampton, MA, USA).

#### 2.3.1. Dynamic Light Scattering

The samples to be examined were transferred to dust-free cylindrical glass cuvettes. DLS measurements were performed using a wide-angle ALV GmbH CGS-3 compact goniometer system, with an avalanche photodiode detector. The detector was connected to an ALV/LSE-5003 electronics unit and an ALV-5000/EPP multi-tau digital corrector. Temperature was controlled using a PolyScience 9102 bath, and in experiments involving temperature variations, the samples were allowed to equilibrate for 20 min before measurement. Each measurement was recorded for 30 s at two scattering angles (90° and 135°). As the results obtained at 90° were found to be more representative, only these are presented. Data analysis was performed with the ALV-Correlator Version 3.0 software. Dynamic light scattering (DLS) measurements of the protein solution produced correlation functions that resulted in multiple peaks corresponding to large-size populations. These signals are likely attributable to transient species or aggregates. As these features were not considered representative of the main protein population, they were excluded from further analysis. Instead, the hydrodynamic radius (R_h_) and polydispersity index (PDI) values were determined using the cumulant method, which provides a more stable description of the predominant species in solution. It is mentioned that the R_h_ of a particle is related to its diffusional properties in solution, and it is equal to the R_h_ of a hypothetical spherical particle with the same diffusion coefficient. Finally, the measured scattered intensity values exhibited a standard deviation of approximately 1–2%, while the corresponding standard deviation for the calculated hydrodynamic radius R_h_ and PDI was approximately 5%.

#### 2.3.2. Fluorescence Spectroscopy

Fluorescence spectroscopy studies were performed for sLOX1 purified samples at a concentration of 0.1 mg/mL in the presence of the three selected buffers using Fluorolog-3, model FL3-21 spectrophotometer, Jobin Yvon-Spex, Horiba Ltd., Kyoto, Japan. The excitation wavelength used was 295 nm, and the emission spectra were recorded in the range of 315 to 500 nm with a 1 nm increment and an integration time equal to 0.5 s. The intrinsic fluorescence of sLOX1 was determined in the presence of all three buffers at 25 °C before (b.h.) and after heating at 75 °C (a.h.). The sLOX1 fluorescence was also measured in the presence of 5 μM thioflavin (ThT), a benzothiazole-based fluorophore, as described previously [34,35], using an excitation wavelength at 440 nm, and the emission spectra were recorded from 300 to 470 nm with an increment of 1 nm and an integration time of 0.5 s. The propensity of the purified sLOX1 solutions to form amyloid structures was explored with the aid of ThT, which was used as an internal probe. A mixture containing the purified enzyme sample at a final concentration of 0.01 mg/mL and 5 μM ThT was preheated at increasing temperatures ranging from 25 °C to 75 °C with a 10 °C increment and measured at each temperature. These measurements were repeated for all the buffers examined.

#### 2.3.3. Electrophoretic Light Scattering

Electrophoretic light scattering measurements were performed using the Zetasizer Nano-ZS (Malvern Panalytical Ltd., Malvern, UK) and a capillary cell appropriate for Zetasizer Nano. All sLOX1 samples in the presence of the three selected buffers were examined before and after heating at 75 °C. The samples (200 μL each) at a concentration of 0.1 mg/mL were injected into the capillary cells carefully to avoid air bubbles. The calculation of zeta potential, $\zeta_{p}$, was performed by using the electrophoretic mobility defined by Henry’s law (Equation (1)) using the Smoluchowski approximation (Equation (2))

(1) $$U=\frac{u}{E},$$

(2) $$\zeta_{p}=\frac{u\eta }{\varepsilon E},$$

where u is the velocity, η the solvent viscosity, ε the dielectric constant, and E the electric field.

#### 2.3.4. Fourier Τransform Ιnfrared Spectroscopy

FTIR was performed using a mid-infrared FTIR spectrometer (Bruker GmbH Equinox 55, Ettlingen, Germany), which was equipped with an attenuated total reflectance (ATR) accessory with spectral range 550–5500 cm^−1^. Background spectrum was collected by recording the clean and dry ATR diamond crystal surface in ambient air. The measurements were performed by creating a thin layer of the sample’s components on the ATR diamond crystal, formed by evaporation of the solvent with the help of a stream of nitrogen. Before each measurement, an aliquot of approximately 200 μL of each sample was placed on the ATR crystal, and the solvent was evaporated. For each sample, the final spectrum is an average of two 100-scan measurements at a 2 cm^−1^ resolution.

### 2.4. Use of Generative Artificial Intelligence (GenAI)

Grammarly (version v1.2.220.1800) AI writing assistance tool, OpenAI. (2023). ChatGPT (5.2 version) [Large language model] and MaxAI browser extension, your AI assistant (v8.35.1), have been used during the preparation of the manuscript for text editing purposes, such as grammar, spelling, punctuation, or language improvement.

## 3. Results

### 3.1. sLOX1 Extraction, Pretreatment, and Purification

**Figure 3:**
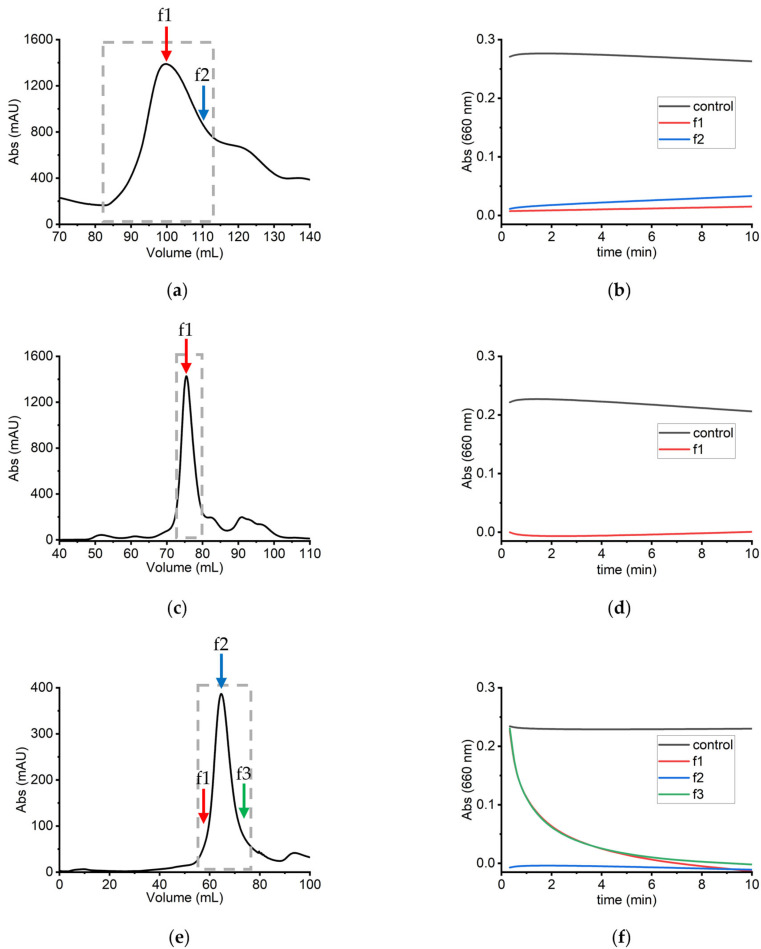
(**a**) The chromatogram of the defatted protein sample following the first DEAE Sepharose column is presented. Arrows indicate the pooled fractions; the boxed region corresponds to those identified as sLOX1-positive. (**b**) Absorbance profiles for the control (no enzyme) and fractions f1 (100 mL) and f2 (110 mL). (**c**) The chromatogram of the eluted sample further purified using a Superdex 200 pg SEC column. Boxed fractions were identified as sLOX1-positive based on colourimetric and SDS-PAGE analyses. (**d**) The time-dependent absorbance profiles for the control and fraction f1 (77.5 mL). (**e**) The chromatogram of the SEC-purified sample following the second DEAE Sepharose column is presented. Arrows and boxed regions indicate the analysed and sLOX1-positive fractions, respectively. (**f**) Absorbance profiles over time for the control and fractions f1 (58 mL), f2 (64 mL), and f3 (74 mL).

The extraction, pretreatment, and purification of sLOX1 were performed using a modified protocol that was first described by Axelrod and coworkers [25], using a DEAE Sepharose column twice, loading 5 times less sample volume than the one previously described, and introducing a high-resolution size exclusion chromatography column as an intermediate step. The modified sample preparation process reduced the required time by half and increased the yield by 60%, resulting in 64 mg sLOX1 from 100 g defatted soybean flour. The quality of sLOX1 was also improved since low molecular weight proteins such as trypsin-like proteases that normally exist in soybean seeds and are responsible for protein degradation were removed [36] (Figure 3b). Fractions collected from each chromatographic step were analysed for sLOX1 activity using a selective colourimetric assay (Section 2.2), and the presence of sLOX1 was verified by SDS-PAGE. Fractions identified as containing sLOX1 were pooled and subjected to subsequent purification steps (Figure 3). Owing to the high enzyme concentration, the colourimetric reaction proceeded rapidly, reaching completion within seconds; consequently, only the initial absorbance trends are shown (Figure 3a,b). The chromatographs and the colourimetric assays were used as criteria to provide conclusive evidence, in addition to SDS-PAGE, for the selection of the fractions containing sLOX1, considering that the three isoforms have very similar molecular weight (Figure 3 and Figures S1–S3).

**Figure 4:**
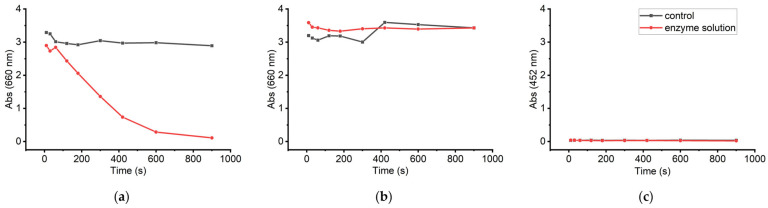
Time-dependent absorbance profiles for the detection of (**a**) sLOX1 at 660 nm in 100 mM sodium borate buffer (pH 9.0), (**b**) sLOX2 at 660 nm in 80 mM sodium phosphate buffer (pH 6.8), and (**c**) sLOX3 at 452 nm in 100 mM sodium phosphate buffer (pH 6.6), using the corresponding specific colorimetric assays for each isoform. Black curves represent control reactions without enzyme, while red curves correspond to reactions containing the purified protein samples.

The presence of sLOX1 isoforms was examined using specific colourimetric assays [33] that detect each specific isoform separately. As it becomes evident from Figure 4, sLOX1 is present and active in the purified sample, whereas the isoforms sLOX2 and sLOX3 are either absent or exist in negligible amounts that cannot be detected through the colourimetric assays.

**Figure 5:**
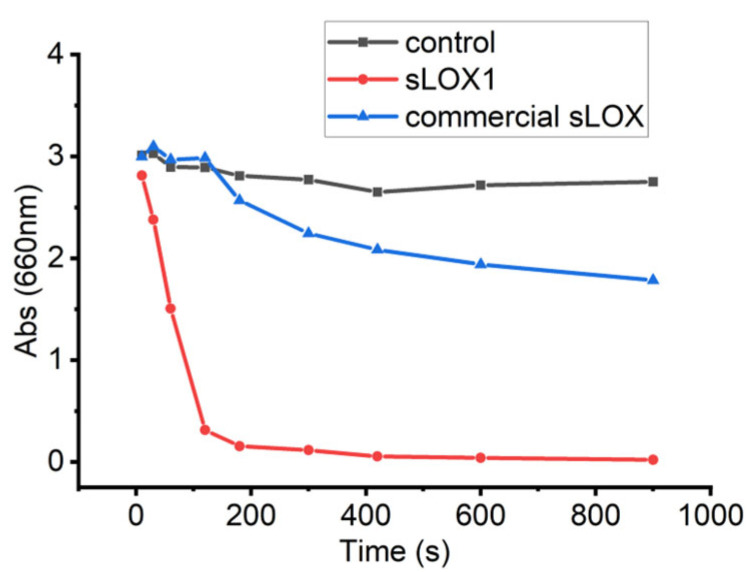
Colourimetric assay results for sLOX1 detection. Absorbance changes are shown for the control (black), the extracted sLOX1 sample (red), and the commercial sLOX enzyme (blue).

The activity of the extracted sample was compared to that of the commercial soybean lipoxygenase. For both cases, samples of 0.25 mg/mL were tested using the colourimetric assay, which is selective for sLOX1 activity. The extracted sLOX1 exhibited a 6-fold faster decrease in absorbance (ΔAbs) compared to the commercial sLOX within the first three minutes (ΔAbs = 2.8). Also, in the case of extracted sLOX1, the reaction reached a plateau, thereafter, indicating complete substrate consumption. By contrast, the commercial sLOX showed a slower reaction rate, with a ΔAbs of 1.2 within the same time period (Figure 5).

The colourimetric assay results for the detection of sLOX1 show that the extracted sLOX1 is more active than the commercial enzyme, providing sufficient indication that the extracted enzyme can be exploited for industrial applications.

### 3.2. Biophysical Characterisation of sLOX1

The biophysical characterisation of the extracted sLOX1 was performed in the presence of the three selected buffers: sodium acetate 0.2 M, pH 4.6, sodium phosphate 0.02 M, pH 6.8, and sodium borate 0.2 M, pH 9.0. The selection of the sodium borate and sodium acetate buffers was made according to previously reported studies for the kinetic and structural characterisation of the enzyme, respectively [20,33,37], while the sodium phosphate buffer was used for the sLOX1 purification. Moreover, the selection of the specific buffers allows the investigation of the effect of pH on the biophysical properties of sLOX1, covering representative values of the whole pH range (i.e., acidic, neutral, and alkaline). The results from the biophysical characterisation studies in the presence of the individual buffers are presented below.

#### 3.2.1. Sodium Borate Buffer 0.2 M pH 9.0

**Figure 6:**
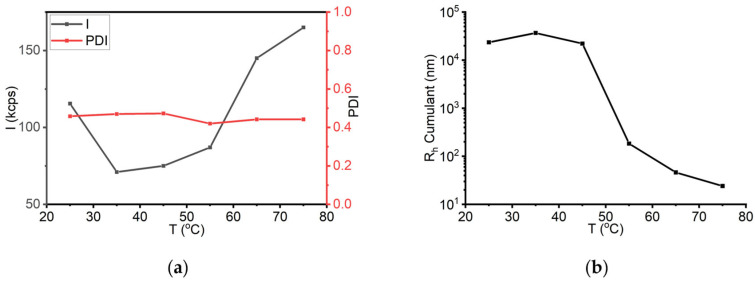
(**a**) DLS scattering intensity (*I*) and PDI, and (**b**) R_h_ Cumulant as a function of temperature for sLOX1 0.1 mg/mL in 0.2 M sodium borate buffer pH 9.0.

sLOX1 solution behaviour was initially evaluated in 0.2 M sodium borate buffer, pH 9.0, a condition under which the enzyme exhibits its highest catalytic activity, according to Siedow, 1991 [38]. Initially, the stability of the 0.1 mg/mL sLOX1 against temperature was examined. For this reason, DLS measurements were performed at a temperature range from 25 to 75 °C by an increase step of 10 °C. The scattered intensity at 25 °C was 115 kcps; it decreased to 71 kcps at 35 °C, and it increased to the final intensity of 165 kcps at 75 °C (Figure 6a). The increase was more intense from 55 to 75 °C. Although there was an increase in intensity, the noticed difference was not significant, considering that the increase in temperature was quite large. sLOX1 seems to be stable enough in this buffer despite heating. The PDI corresponded to a value of about 0.45 through the increase of temperature, which can also be considered as an indication of the solution’s stability (Figure 6a). At the same time, the hydrodynamic radius R_h_ (derived from Cumulant analysis) ranged from 25 to 35 μm at the onset of the heat treatment. When the temperature reached 55 °C and above, the R_h_ significantly decreased to values ranging from 180 to 25 nm (Figure 6b). This finding supports the hypothesis that elevated temperature facilitates aggregate dissociation and contributes to the stabilization of the system.

**Figure 7:**
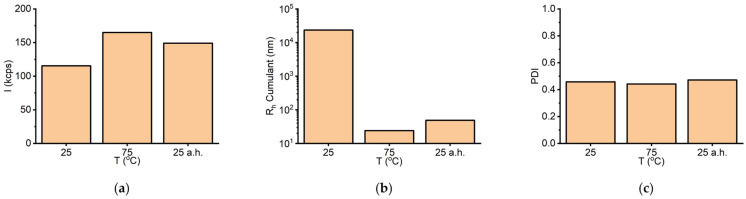
(**a**) DLS scattering intensity (*I*), (**b**) R_h_ Cumulant, and (**c**) PDI at room temperature (25 °C), at the highest temperature investigated (75 °C), and at room temperature after heating (25 °C a.h.) for sLOX1 0.1 mg/mL in 0.2 M sodium borate buffer pH 9.0.

As the DLS data indicated that no significant biophysical changes occurred above 55 °C, further experiments were performed to investigate the reversibility of thermal effects on the enzyme solution. Specifically, the sample was analysed at room temperature (25 °C), then heated to 75 °C, and finally cooled back to 25 °C before a final measurement. The main purpose of these measurements was to check the stability before and after heating (marked as b.h. and a.h., respectively). The scattering intensity increased from 115 kcps (initial) to 165 kcps upon heating and subsequently decreased to 149 kcps after the sample returned to room temperature 1 h later (Figure 7a). On the other hand, the size (as expressed by the corresponding R_h_ Cumulant values) remained rather low (approximately 45 nm) upon returning to 25 °C following heat treatment (Figure 7b). Moreover, the PDI value persisted at ~0.45 (Figure 7c), similar to the corresponding values recorded during heating. Taken together, these results suggest that the thermal effect on sLOX1 in sodium borate buffer (pH 9.0) does not induce substantial changes in the enzyme’s solution behaviour. Nevertheless, it is possible that bigger aggregates either dissociated or precipitated after the heating procedure, as indicated by the observed decrease of R_h_.

Electrophoretic light scattering measurements were also performed under the same conditions. At room temperature, the *ζ* potential was measured at −7 mV. After heating to 75 °C and subsequent cooling back to room temperature, the zeta potential decreased to −18 mV. This increase in absolute *ζ*_p_ value may be attributed to conformational changes in the protein structure upon heating. The relatively high (in terms of absolute value) negative *ζ* potential indicates that the system remains quite stable. In combination with the DLS results, these findings indicate that sLOX1 undergoes some minor structural alterations during thermal exposure.

**Figure 8:**
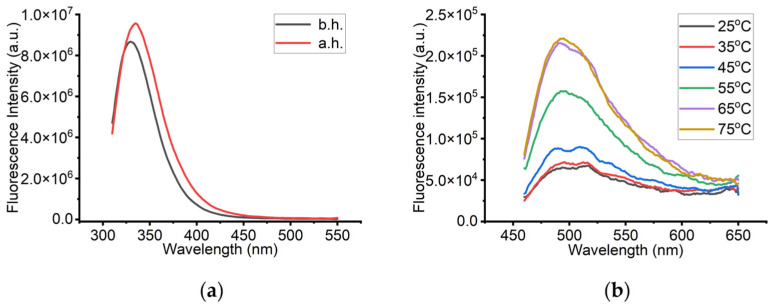
(**a**) Tryptophan fluorescence spectra of a 0.1 mg/mL sLOX1 solution in 0.2 M sodium borate buffer at pH 9.0, before (b.h.) and after heating (a.h.), and (**b**) Thioflavin T (ThT) fluorescence spectra in the presence of 0.1 mg/mL sLOX1 in 0.2 M sodium borate buffer of pH 9.0 during heating.

To further evaluate the enzyme’s structural integrity under thermal stress, fluorescence spectroscopy was employed. At room temperature, the emission maximum was observed at 331 nm (Figure 8a). After heating and cooling back to 25 °C, the emission maximum showed a slight red shift to 333 nm, accompanied by a ~5% increase in fluorescence intensity (Figure 8a). This small shift may be indicative of increased hydrophobic exposure, possibly due to a different environment around aromatic residues—particularly tryptophan—resulting from thermal conformational rearrangement.

In parallel, ThT fluorescence measurements were conducted to investigate potential amyloid formation. sLOX1 samples after addition of ThT were measured from 25 to 75 °C in 10 °C increments. As shown in Figure 8b, the emission maximum remained constant across all temperatures; however, fluorescence intensity increased progressively up to 65 °C. No significant change was observed between 65 and 75 °C. Since ThT specifically binds to *β*-sheet-rich structures and amyloid fibrils, this increase in signal likely reflects the formation of amyloid-like aggregates as the temperature increased. The plateau at 75 °C may indicate that fibril formation reached completion by 65 °C, with no additional structural changes thereafter. The corresponding maximum ThT fluorescence intensities for these experiments are summarized in Figure S19, providing an overview of the aggregation behaviour under the tested conditions. To gain additional insights into possible secondary structural alterations, FTIR spectroscopy was performed. Representative spectra were collected for two samples of 0.1 mg/mL sLOX1 in 0.2 M sodium borate buffer, pH 9.0: one maintained at room temperature (b.h.), and another subjected to heating at 75 °C followed by cooling to 25 °C (a.h.). However, the FTIR spectra did not clearly resolve the characteristic amide I and II regions, typically associated with protein backbone vibrations. This limitation is likely due to the high buffer concentration relative to the protein concentration, which may have masked the protein-specific signals (Figure S4).

**Figure 9:**
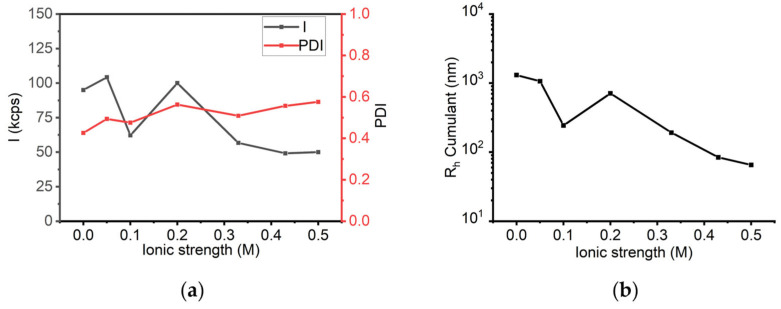
(**a**) DLS scattering intensity (*I*) and PDI, and (**b**) R_h_ Cumulant as a function of ionic strength (NaCl) for sLOX1 0.1 mg/mL in 0.2 M sodium borate buffer, pH 9.0.

Subsequent DLS measurements were conducted to evaluate the stability of sLOX1 under increasing ionic strength. This was achieved by incrementally adding sodium chloride into the enzyme solution at concentrations ranging from 0 to 0.5 M. The initial scattered intensity of the solution in the absence of sodium chloride was 95 kcps (Figure 9a). As the sodium chloride concentration increased, a slight deviation of the intensity was observed, reaching 50 kcps at 0.5 M. The PDI values ranged from 0.4 to 0.6 across the tested salt concentrations, indicating moderate sample heterogeneity (Figure 9a). Cumulant analysis revealed that the average hydrodynamic radius (R_h_) decreased upon addition of salt. In the absence of NaCl, the average R_h_ was approximately 13 μm, while higher salt concentrations promoted a rather gradual reduction in particle size (Figure 9b), reaching values below 100 nm at the highest salt content. These results suggest that the increase of ionic strength may contribute to the overall system stabilization as it promotes the dissociation of populations with larger sizes.

**Figure 10:**
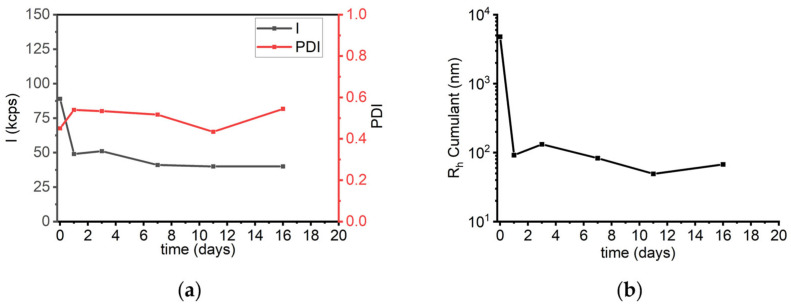
(**a**) DLS scattering intensity (*I*) and PDI, and (**b**) R_h_ Cumulant as a function of time (days) for sLOX1 0.1 mg/mL in 0.2 M sodium borate buffer pH 9.0.

To assess long-term stability, sLOX1 in 0.2 M sodium borate buffer (pH 9.0) was monitored over 16 days via DLS. The solution was measured by DLS at different intervals over the course of time, as shown in Figure 10. On the day of preparation, the scattering intensity was 89 kcps; it declined to 51 kcps on day 2 and eventually reached 40 kcps by day 16. Although a decrease in intensity was observed, the variation remained relatively small (Figure 10a). Throughout the time course, PDI values remained in the range of 0.4 to 0.6, indicating no significant changes in the polydispersity of the solution (Figure 10a). At the same time, the R_h_ (Cumulant analysis) initially measured at 4.7 μm decreased to 90 nm by day 2, after which it remained relatively stable (Figure 10b). These observations suggest that sLOX1 remains stable in sodium borate buffer over time, both in terms of scattering behaviour and particle size distribution. In addition, larger populations most probably dissociate, also contributing to the temporal stability of the enzyme.

#### 3.2.2. Sodium Phosphate Buffer 0.02 M pH 6.8

Similar DLS measurements were also performed for sLOX1 in sodium phosphate buffer 0.02 M, pH 6.8, to investigate the influence of buffer composition on protein oligomerization and structural behaviour. The experimental conditions were kept consistent with those used for the sodium borate buffer.

Temperature-dependent DLS measurements were carried out by gradually increasing the temperature from 25 to 75 °C, with a 10 °C increment. In the case of the phosphate buffer, two different concentrations of 0.1 mg/mL and 1 mg/mL were used. The solution of 0.1 mg/mL was investigated to compare the behaviour of the enzyme in sodium phosphate buffer and in sodium borate buffer. At the same time, the solution of 1 mg/mL was used to investigate the behaviour of the enzyme in higher concentrations, which would be used at industrial applications. For the 0.1 mg/mL sLOX1 solution, a notable increase in scattering intensity was observed at temperatures above 65 °C. In a similar manner, the 1 mg/mL solution exhibited a substantial increase in scattering intensity at temperatures above 55 °C, with the signal increasing approximately 44-fold relative to the lower concentration (Figure S5). This suggests that higher enzyme concentration favours a more pronounced aggregation at elevated temperatures. PDI remained within the range of 0.4 to 0.5 for both concentrations at temperatures below 65 °C (Figure S5). At 0.1 mg/mL, PDI values decreased above 65 °C, possibly due to the formation of aggregates with a more uniform size distribution. In parallel, for the 1 mg/mL solution, PDI decreased at 65 °C but increased again at 75 °C. This behaviour indicates the emergence of heterogeneous aggregate populations, consistent with changes observed in the corresponding Cumulant R_h_ values (Figure S6). Specifically, for both concentrations, average R_h_ increased with temperature up to 45 °C, followed by a decline at higher temperatures. This pattern suggests that although aggregation is enhanced at elevated temperatures, larger aggregates may precipitate, thereby reducing the average R_h_ of the remaining particles in solution. Notably, the R_h_ value of 6.5 μm measured at 75 °C for the 1 mg/mL solution further supports this interpretation, pointing to the formation of large aggregates that may sediment over time. Altogether, these findings highlight that enzyme concentration significantly modulates the extent and nature of heat-induced oligomerization and aggregation in phosphate buffer.

**Figure 11:**
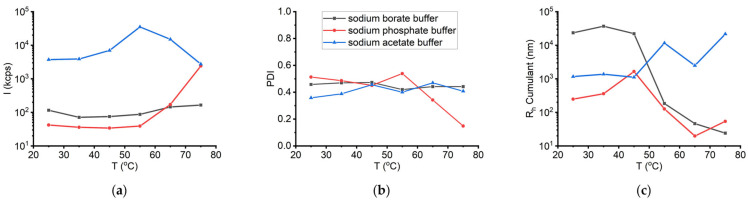
(**a**) DLS scattering intensity (*I*), (**b**) PDI, and (**c**) R_h_ Cumulant as a function of temperature for sLOX1 0.1 mg/mL in 0.2 M sodium borate buffer, pH 9.0 (black line), in 0.02 M sodium phosphate buffer, pH 6.8 (red line), and in 0.2 M sodium acetate buffer, pH 4.6 (blue line).

To facilitate the direct comparison of the effect of temperature on the physicochemical properties of the enzyme under the different buffer conditions, Figure 11 presents the sum of the obtained DLS results for the sLOX1 solutions in the three different investigated buffers. In contrast to the results obtained in sodium borate buffer, the presence of sLOX1 in sodium phosphate buffer appears to promote oligomerization/aggregation at both low (0.1 mg/mL) and high (1 mg/mL) enzyme concentrations. As observed, temperature increase leads to a notable increase in scattering intensity above 65 °C for the 0.1 mg/mL solution and above 55 °C for the 1 mg/mL solution.

**Figure 12:**
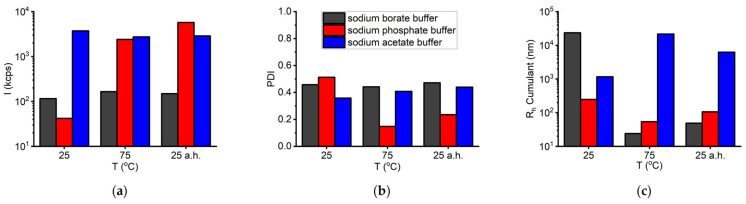
(**a**) DLS scattering intensity (*I*), (**b**) PDI, and (**c**) R_h_ Cumulant at room temperature (25 °C), at the highest temperature investigated (75 °C), and at room temperature after heating (25 °C a.h.) for sLOX1 0.1 mg/mL in 0.2 M sodium borate buffer, pH 9.0 (black), in 0.02 M sodium phosphate buffer, pH 6.8 (red), and in 0.2 M sodium acetate buffer, pH 4.6 (blue).

To further investigate the effect of heat on protein aggregation, DLS measurements were performed at 25 °C (b.h.), at 75 °C, and again at 25 °C after the sample was allowed to cool (a.h.) (Figure S7). The results revealed that, for both concentrations, scattering intensity increased further after cooling to 25 °C post-heating (Figure S7a). This indicates that the heat-induced aggregation is largely irreversible, with aggregates remaining in solution after the temperature returns to ambient conditions. Although the average R_h_ showed a slight decrease for the 0.1 mg/mL sLOX1 solution (Figure S7b), the change was relatively small. On the other hand, for the 1 mg/mL solution, R_h_ increased even further when the sample returned to ambient conditions after heating. This contrast between the two concentrations indicates that a higher enzyme concentration promotes a more pronounced aggregation upon heating. In the case of 0.1 mg/mL, PDI decreased from 0.5 to 0.15 when the temperature increased to 75 °C, and it subsequently increased to 0.23 when the solution returned to room temperature. On the other hand, for 1 mg/mL sLOX1, PDI exhibited a decrease from 0.5 to 0.4 (Figure S7c) after cooling, further supporting the conclusion that aggregates formed at high concentrations are more stable and persist after heating. By comparing the results obtained for the 0.1 mg/mL enzyme solutions prepared in sodium borate and sodium phosphate buffers (Figure 12), it is evident that the enzyme exhibits distinct behaviour depending on the buffer environment. In sodium borate buffer, the enzyme appeared more stable during heat treatment, suggesting that this buffer exerts a stabilizing effect on the protein conformation. In contrast, in sodium phosphate buffer, the results indicate a tendency toward aggregation. Thus, it seems that this medium may promote intermolecular interactions leading to reduced stability under the same thermal conditions.

Changes in the conformation state of sLOX1 in sodium phosphate buffer were further investigated using ELS. For this purpose, a 0.1 mg/mL sLOX1 solution in this buffer (pH 6.8) was initially examined at room temperature. The zeta potential of the enzyme was measured at −8 mV, indicating a negatively charged surface under these conditions. After the solution was heated at 75 °C and subsequently cooled down to room temperature, the zeta potential decreased to −13 mV. A similar trend was observed for the 1 mg/mL solution, suggesting a concentration-independent effect. This decrease in zeta potential likely reflects heat-induced conformational rearrangements, possibly involving repositioning of amino acids with negatively charged side chains toward the protein surface. This lower *ζ* potential after heating makes the repulsive forces between molecules more intense, which enhances the stability of the system.

To further evaluate structural changes, fluorescence spectroscopy was employed. At 25 °C, the position of the maximum emission was observed at 331 nm (Figure S8a), identical to the result in sodium borate buffer (Figure 8a). However, following heat treatment, the emission maximum shifted to 336 nm, in contrast to the 333 nm shift seen in the borate system. Additionally, a 10% decrease in fluorescence intensity was recorded, as opposed to the 5% increase observed in borate buffer. This reduction may indicate a change in the local environment of tryptophan residues, possibly caused by partial unfolding or rearrangement of the tertiary structure.

Fluorescence measurements in the presence of 5 μM ThT were also performed to monitor potential amyloid formation. At room temperature, the maximum fluorescence emission was observed at 497 nm (Figure S8b). Upon heating to 75 °C, the ThT fluorescence intensity decreased by 56%, while the position of the emission maximum remained unchanged. This decline likely reflects precipitation of larger aggregates rich in *β*-sheet conformations, such as amyloid fibrils, to which ThT binds. These findings are in contrast with the behaviour in sodium borate buffer, where heating led to increased ThT fluorescence. It seems that the nature of aggregation and that of aggregate structure differ between the two buffer systems (Figure S19).

To further explore possible changes in secondary structure and the presence of *β*-sheets or *α*-helices within the enzyme structure, FTIR was employed. In this case, the concentration of the buffer is lower (than in the case of the sodium borate buffer), thus allowing characteristic amide peaks to be discerned. FTIR spectra were obtained for four different samples of sLOX1 in 0.02 M sodium phosphate buffer, pH 6.8 (i.e., both 0.1 and 1 mg/mL at room temperature before and after heating at 75 °C). As shown in Figure S9b, all spectra exhibited a characteristic peak at 1650 cm^−1^, consistent with the Amide I region, which is typically associated with *α*-helical content. A second peak at 1540 cm^−1^, corresponding to the Amide II region, was also detected. In samples containing 0.1 mg/mL sLOX1, the Amide I and II signals were less distinct, due to the lower protein concentration, while higher enzyme concentration (i.e., 1 mg/mL) yielded more defined spectra. Notably, for the 1 mg/mL sample after heating at 75 °C, the shape of the Amide I band changed, showing a significant contribution from an underlying peak located at about 1625–1630 cm^−1^. This contribution is characteristic of *β*-sheets [39,40] and further confirms that the heat-induced aggregation of the enzyme leads to their formation, as also evidenced by the ThT fluorescence measurements.

**Figure 13:**
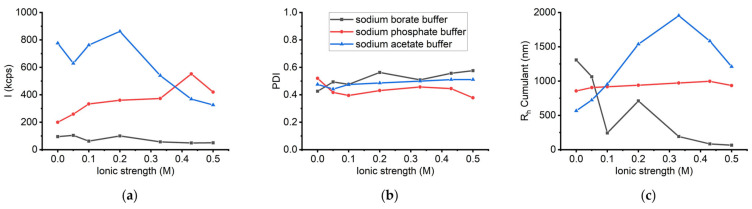
(**a**) DLS scattering intensity (*I*), (**b**) PDI, and (**c**) R_h_ Cumulant as a function of ionic strength (NaCl) for sLOX1 0.1 mg/mL in 0.2 M sodium borate buffer, pH 9.0 (black line), in 0.02 M sodium phosphate buffer, pH 6.8 (red line), and in 0.2 M sodium acetate buffer, pH 4.6 (blue line).

Subsequent DLS experiments were conducted to compare the behaviour of sLOX1 under increased ionic strength in the presence of sodium phosphate buffer, pH 6.8, with the corresponding results obtained in the case of sodium borate buffer. A direct comparison of the obtained results is shown in Figure 13. In the presence of the sodium phosphate buffer, increasing the sodium chloride concentration from 0 to 0.42 M resulted in a substantial rise in scattering intensity from 200 to 550 kcps, representing a 2.75-fold increase (Figure S10a). However, upon further increase of the NaCl concentration to 0.5 M, the intensity declined (Figure S10a). This trend indicates that moderate salt concentrations may promote protein aggregation by shielding surface charges. On the other hand, higher ionic strength (≥0.42 M) could either induce precipitation of larger aggregates or disrupt larger assemblies through destabilizing hydrophobic interactions. In parallel to these variations in intensity, the average hydrodynamic radius (R_h_) showed a small gradual increase—from 850 to 1000 nm—as NaCl concentration increased (Figure S10b), suggesting the persistence of large aggregate populations. Nevertheless, a slight decrease of R_h_ at 0.5 M ionic strength is also observed, consistent with the notion that larger aggregates precipitated. At the same time, the corresponding PDI values ranged from 0.4 to 0.5 throughout the titration (Figure S10a). In contrast to the results obtained with the sodium borate buffer—where increasing sodium chloride content appeared to promote breakdown of larger oligomeric species—the findings in phosphate buffer suggest that elevated ionic strength enhances aggregation and oligomerization of sLOX1.

**Figure 14:**
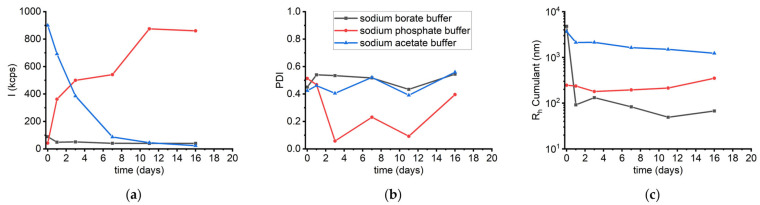
(**a**) DLS scattering intensity (*I*), (**b**) PDI, and (**c**) R_h_ Cumulant as a function of time (days) for sLOX1 0.1 mg/mL in 0.2 M sodium borate buffer, pH 9.0 (black line), in 0.02 M sodium phosphate buffer, pH 6.8 (red line), and in 0.2 M sodium acetate buffer, pH 4.6 (blue line).

A further comparison was made to assess the long-term stability of sLOX1 in the presence of sodium phosphate buffer over a period of 16 days, mirroring the conditions used for borate buffer. The corresponding collective results for all three buffers are given in Figure 14. Over time, the scattering intensity increased progressively from 160 kcps on day 1 to 860 kcps on day 16 (Figure S11a), indicating ongoing aggregation. PDI varied slightly, exhibiting values below 0.5 during this period, while the average R_h_ initially measured at 247 nm remained at values between 200 and 350 nm until the 16th day (Figure S11b). This progressive increase in intensity, along with the small variations in R_h_, is in contrast to the behaviour observed in sodium borate buffer, where the intensity remained relatively constant (~45–50 kcps) (Figure 10a). These findings suggest that in the phosphate buffer, sLOX1 exhibits a higher propensity for time-dependent aggregation, likely leading to the formation of smaller but more numerous aggregate populations.

#### 3.2.3. Sodium Acetate Buffer 0.2 M pH 4.6

The last sLOX1 solution condition, which was tested, contained 0.1 mg/mL sLOX1 in 0.2 M sodium acetate buffer, at pH 4.6. The first DLS measurements implemented for this buffer were related to the influence of temperature (25–75 °C range) on the stability of sLOX1. As shown in Figure S12a, the scattering intensity increased by 9-fold while the temperature increased from 25 to 55 °C. At this temperature, the solution became opaque, as shown in Figure S13. Upon additional increase in temperature, the intensity decreased, most probably due to the precipitation of the larger thermally induced aggregates. In addition, R_h_ (Cumulant) was about 1 μm from 25 to 45 °C, and it increased to 11.6 μm at 55 °C. Upon further heating, the R_h_ initially decreased at 65 °C, which could be related to the presumed precipitation of larger entities in solution, and subsequently increased again, reaching its maximum value at 75 °C, indicating further aggregation (Figure S12b). However, PDI points to a medium polydispersity equal to 0.4 at almost all different temperatures measured (Figure S12a), which suggests a relatively stable distribution of populations in the solution. Still, the fact that the solution became opaque at 55 °C, as well as the overall significantly higher intensity values (Figure 11), supports the assumption of thermally induced aggregation of sLOX1 in this buffer (Figure S13).

As the heating of the solution above 55 °C promoted significant changes, the stability of sLOX1 before and after heating was investigated, as in the case of the other two buffers (Figure 12). When the solution returned to room temperature, the intensity remained approximately at the same value as at 75 °C (Figure S14a), while the average R_h_ decreased by 3.5-fold (Figure S14b). PDI points to medium polydispersity equal to 0.4 both before and after heating (Figure S14c). It is important to mention that the observed heat-induced opaqueness was not reversed when the solution returned to room temperature. In the meantime, during the cooling of the solution back to room temperature, the large aggregates possibly precipitated. This phenomenon could explain the observed decrease in intensity and R_h_ values, in comparison to those at 55 °C.

ELS measurements were also executed for the 0.1 mg/mL enzyme solution in acetate buffer. The *ζ* potential was equal to −13 mV at room temperature, but after heating the solution at 75 °C, the *ζ* potential was equal to 2 mV, which is in contrast to the observed decrease of *ζ* potential observed in the two previous buffers. These results could be explained by the observed intense aggregation and precipitation of the enzyme in this buffer after heating at 75 °C. It seems that the species left in the solution after the thermal treatment exhibit rather low charge, which is directly related to their reduced solubility/stability.

Taking into consideration all the results from measurements correlated to the heat treatment of sLOX1 solutions in the various buffers, it can be inferred that the increase of temperature promotes the aggregation of the enzyme in the presence of sodium acetate buffer more intensely than in the case of sodium borate and sodium phosphate buffer (Figure 11 and Figure 12).

The thermal stability of the protein solution in the presence of sodium acetate buffer was also examined by fluorescence spectroscopy. As presented in Figure S15a, the intensity maximum was located at 328 nm at 25 °C and was shifted to 332 nm after heating at 75 °C. This represents a 4 nm difference compared to the values observed in sodium borate and sodium acetate buffers. In addition, the fluorescence intensity decreased by 13% before and after heating. By comparing these results with those from the other two buffers (see Figure 8a and Figure S8a), it is shown that only in the case of the sodium borate buffer an increase in fluorescence intensity was observed, while in the presence of the two other buffers, the intensity decreased.

Fluorescence measurements were also performed in the presence of ThT in the sLOX1 solution and the 25–75 °C temperature range. As shown in Figure S15b, the fluorescence intensity increased with the increase of temperature from 25 to 65 °C. Nevertheless, a further increase in temperature to 75 °C led to a remarkable decrease in intensity. This decrease could be explained by a potential increase in amyloid fibril formation and their subsequent extended precipitation. The influence of buffer composition on the extent of aggregation is further illustrated by the comparative ThT fluorescence data presented in Figure S19.

FTIR measurements were also performed for two different samples of sLOX1 in the presence of sodium acetate buffer and 0.1 mg/mL concentration. That is both at room temperature as well as after the heating of the sample at 75 °C and its cooling back to room temperature. The results of these measurements failed to give significant information about the secondary structure of the enzyme, as was also the case for the sodium borate buffer (Figure S16), most probably due to the high concentration of the buffer. Nevertheless, this buffer was examined because sodium acetate was previously used in crystallization studies [20], and the measurements were performed under identical conditions to allow direct comparison across methods, as discussed for the sodium borate buffer.

The next set of DLS measurements aimed to evaluate the influence of ionic strength on the stability of the enzyme in the given buffer system. The scattering intensity increased by approximately 50% when the sodium chloride concentration increased from 0.05 to 0.2 M (Figure S17a). However, further increase in ionic strength led to a decline in scattering intensity. Concurrently, the average R_h_ was increased by 4-fold as the NaCl concentration reached 0.3 M, but then decreased to nearly half its peak value upon further salt additions (Figure S17b). This behaviour—an initial increase in scattering intensity followed by a decrease—can be attributed to the aggregation of sLOX1 in the presence of moderate salt concentrations, followed by precipitation of larger aggregates as ionic strength surpassed 0.2 M. The observed changes in R_h_ Cumulant values support this interpretation: R_h_ increased during aggregation but subsequently decreased as aggregates precipitated out of solution. Additionally, the PDI remained at approximately 0.4 (Figure S17a). These trends in intensity and R_h_ align with those observed in sodium phosphate buffer, demonstrating the tendency of sLOX1 to aggregate under elevated ionic strength in both buffer systems (Figure 13).

A final series of DLS measurements was conducted to assess the long-term stability of sLOX1 in this buffer over time. Measurements were taken at consistent time intervals, in analogy to the other two buffer systems. Initially, the scattering intensity was measured at 900 kcps on the day the sample was prepared, and it progressively decreased over time, reaching just 23 kcps by day 16 (Figure S18a). This substantial decline likely reflects the gradual precipitation of sLOX1. This hypothesis is further supported by the R_h_ Cumulant values, which exhibited a small, gradual decrease over time (Figure S18b). In parallel, PDI values ranged from 0.4 to 0.6, which indicates a rather heterogeneous distribution of particle sizes (Figure S18a). These findings are similar to those observed in sodium phosphate buffer, but they are in contrast with the stability observed in sodium borate buffer, where both intensity and R_h_ remained relatively stable over time (Figure 14).

## 4. Discussion

The present work provides a detailed biophysical investigation of sLOX1, focusing on the influence of buffer composition, temperature and ionic strength on its structural stability and aggregation behaviour. Specifically, the enzyme retains its structural integrity and functional properties during heat treatment up to at least 55 °C. Furthermore, sLOX1 displays reduced aggregation tendencies both over time and under increasing ionic strength conditions when sodium borate buffer is used, suggesting that this buffer provides a stabilizing environment for the enzyme. At the same time, sLOX1 in sodium phosphate buffer (pH 6.8) exhibits distinct signs of thermally induced aggregation above 55 °C but rather relative stability against ionic strength and time. On the other hand, it is observed that sLOX1 in sodium acetate buffer (pH 4.6) has a high tendency to aggregate against all examined destabilizing factors (i.e., temperature, ionic strength, and time). This behaviour is possibly associated with the acidic environment of the solution, as the pH is below the isoelectric point (pI) of sLOX1 (i.e., pI = 5.96 as determined through the Expasy portal [41]), a condition that is generally unfavourable for maintaining the stability of the enzyme. Under conditions that promoted aggregation, the aggregation of sLOX1 was found to be predominantly irreversible across all three buffer systems, with this effect being more pronounced in sodium phosphate and sodium acetate buffers. No evidence of aggregate re-dissolution or recovery of the initial hydrodynamic size distribution was observed upon further variation of temperature, ionic strength, or incubation time. Any apparent decreases in scattering intensity or R_h_ Cumulant are therefore attributed to sedimentation or precipitation of larger aggregates rather than to partial reversibility of the aggregation process. Although light scattering techniques constituted the primary tools for probing aggregation and colloidal stability, complementary FTIR and fluorescence spectroscopy, including ThT assays, were employed to provide additional insight into temperature-induced structural alterations of sLOX1. Together, these approaches enabled a coherent interpretation of aggregation phenomena and associated conformational changes within the scope of an initial, systematic investigation. A comparative overview of the observed stability trends across the three buffer systems is provided in Table 1, summarizing the distinct effects of temperature, incubation time, and ionic strength on sLOX1 behaviour.

The proposed isolation and characterization protocol was optimized at laboratory scale, and the buffer systems employed were selected primarily for experimental control and mechanistic insights, in compliance with previously reported studies, rather than to fully replicate the physiological conditions in the plant. However, the use of commonly available buffers and chromatographic materials and the enzyme stability that was observed under industrially relevant conditions provide sufficient evidence to support potential scalability. The cost efficiency and throughput at an industrial scale have yet to be explored to optimize further the purification process (e.g., chromatography matrix effects), and explore alternative buffering systems that maintain the required pH for separation while improving enzyme stability.

## 5. Conclusions

The extraction and purification of sLOX1 using the described protocol resulted in an enzyme preparation with significantly higher activity compared to commercially available lipoxygenase. Comprehensive biophysical characterization demonstrated that sLOX1 exhibits remarkable stability, particularly in the presence of sodium borate buffer (pH 9). Overall, this study provides one of the most comprehensive characterizations of sLOX1, examining its behaviour across diverse buffer systems, temperature conditions, and ionic strengths, employing several complementary techniques. The purified isoform was shown to have high efficiency and purity, properties that render it a promising candidate for use as a biocatalyst in the production of industrially relevant intermediates. Furthermore, the detailed biophysical characterisation allows for a deeper understanding of sLOX1 behaviour in aqueous media, which is directly related to sLOX1 quality as a biocatalyst.

## Figures and Tables

**Table 1:** Comparative summary of the thermal stability, aggregation behaviour, long-term stability, and ionic strength sensitivity of sLOX1 in the three buffer systems examined.

Buffer	Thermal Stability and Aggregation Behaviour	Long-Term Stability	Ionic Strength Sensitivity
sodium borate 0.2 M, pH 9.0	quite stable (up to 75 °C)	quite stable (up to 16 days)	quite stable (up to 0.5 M NaCl)
sodium phosphate 0.02 M, pH 6.8	quite stable (up to 65 °C)	time-dependent aggregation	tendency for aggregation (≥0.42 M NaCl, potential precipitation)
sodium acetate 0.2 M, pH 4.6	thermally induced aggregation (≥55 °C precipitation)	time-dependent aggregation and potential precipitation	time-dependent aggregation (≥0.20 M NaCl, potential precipitation)

## Data Availability

Data are contained within the article and Supplementary Materials.

## Supplementary Materials

The following supporting information can be downloaded at: https://www.mdpi.com/article/10.3390/biom16010162/s1, Figure S1: SDS PAGE gel of the fractions after the first DEAE; Figure S2: SDS PAGE gel of the fractions after the SEC; Figure S3: SDS PAGE gel of the purified sample; Figure S4: FTIR absorbance spectra of sLOX1 in 0.2 M sodium borate buffer, pH 9.0; Figure S5: DLS scattering intensity (*I*) and PDI as a function of temperature for sLOX1 in 0.02 M sodium phosphate buffer, pH 6.8; Figure S6: R_h_ Cumulant as a function of temperature for sLOX1 in 0.02 M sodium phosphate buffer, pH 6.8; Figure S7: DLS scattering intensity (*I*), R_h_ Cumulant, and PDI at different temperatures for sLOX1 in 0.02 M sodium phosphate buffer, pH 6.8; Figure S8: Tryptophan and Thioflavin T (ThT) fluorescence spectra in the presence of sLOX1 in 0.02 M sodium phosphate buffer, pH 6.8; Figure S9: FTIR absorbance spectra of sLOX1 in 0.02 M sodium phosphate buffer, pH 6.8; Figure S10: DLS scattering intensity (*I*), PDI, and R_h_ Cumulant values as a function of ionic strength (NaCl) for sLOX1 0.1 mg/mL in 0.02 M sodium phosphate buffer, pH 6.8; Figure S11: DLS scattering intensity (*I*), PDI, and R_h_ Cumulant as a function of time (days) for sLOX1 0.1 mg/mL in 0.02 M sodium phosphate buffer, pH 6.8; Figure S12: DLS scattering intensity (*I*), PDI, and the R_h_ Cumulant as a function of temperature for sLOX1 0.1 mg/mL in 0.2 M sodium acetate buffer, pH 4.6; Figure S13: Precipitates present in 0.1 mg/mL sLOX1 solution in 0.2 M acetate buffer, pH 4.6 at 55 °C; Figure S14: DLS scattering intensity (*I*), R_h_ Cumulant, and PDI at different temperatures for sLOX1 0.1 mg/mL in 0.2 M sodium acetate buffer, pH 4.6; Figure S15: Tryptophan and Thioflavin T (ThT) fluorescence spectra in the presence of 0.1 mg/mL sLOX1 in 0.2 M sodium acetate buffer, pH 4.6; Figure S16: FTIR absorbance spectra of sLOX1 in 0.2 M sodium acetate buffer, pH 4.6; Figure S17: DLS scattering intensity (*I*), PDI, and the R_h_ Cumulant as a function of ionic strength (NaCl) for sLOX1 0.1 mg/mL in 0.2 M sodium acetate buffer, pH 4.6; Figure S18: DLS scattering intensity (*I*), PDI, and the R_h_ Contin as a function of time (days) for sLOX1 0.1 mg/mL in 0.2 M sodium acetate buffer, pH 4.6; Figure S19: Maximum fluorescence intensity as a function of temperature in the presence of ThT for sLOX1 0.1 mg/mL in 0.2 M sodium borate buffer, pH 9.0.

## Abbreviations

The following abbreviations are used in this manuscript:

ATR	Attenuated total reflectance
CD	Circular Dichroism
DLS	Dynamic Light Scattering
DSC	Differential Scanning Calorimetry
ELS	Electrophoretic Light Scattering
EPR	Electron Paramagnetic Resonance
FTIR	Fourier Transform Infrared
HETE	Hydroxy-eicosatetraenoic acid
ITC	Isothermal Titration Calorimetry
LA	Linoleic Acid
LT	Leukotriene
LOX	Lipoxygenase
PDI	Polydispersity Index
R_h_	Hydrodynamic radius
R.m.s.d.	Root mean square deviation
SEC	Size Exclusion Chromatography
sLOX1	Soybean lipoxygenase-1
SPI	soybean protein isolate
ThT	Thioflavin T
