# Supplementary material for: Isolation and Biophysical Characterization of Lipoxygenase-1 from Soybean Seed, a Versatile Biocatalyst for Industrial Applications
Source: Biomolecules. 2026 Jan 19;16(1):162. doi: 10.3390/biom16010162 (PMC12838591; doi:10.3390/biom16010162)
Supplement: Supplementary file 1 [file biomolecules-16-00162-s001.zip › biomolecules-4101185-supplementary.pdf]

biomolecules

Article

# Isolation and biophysical characterization of lipoxygenase-1 from soybean seed, a versatile biocatalyst for industrial applications

**Ioanna Gerogianni [1,3], Antiopi Vardaxi [2], Ilias Matis [1], Maria Karayianni [2], Maria Zoumpanioti [1], Thomas Mavromoustakos [3], Stergios Pispas [2,*] and Evangelia D. Chrysina [1,*]**

[1] Institute of Chemical Biology, National Hellenic Research Foundation, 48 Vassileos Constantinou Ave., Athens 116 35, Greece
[2] Theoretical and Physical Chemistry Institute, National Hellenic Research Foundation, 48 Vassileos Constantinou Ave., Athens 116 35, Greece
[3] Department of Chemistry, National and Kapodistrian University of Athens, Panepistimioupolis Zografou 15772, Greece

* Correspondence: pispas@eie.gr, echrysina@eie.gr

## Supplementary Materials

The Supplementary Materials include photos of the SDS-PAGE gels used during the enzyme purification procedure, as well as additional, DLS, ELS, fluorescence and FTIR data obtained in sodium borate, sodium phosphate and sodium acetate buffers.

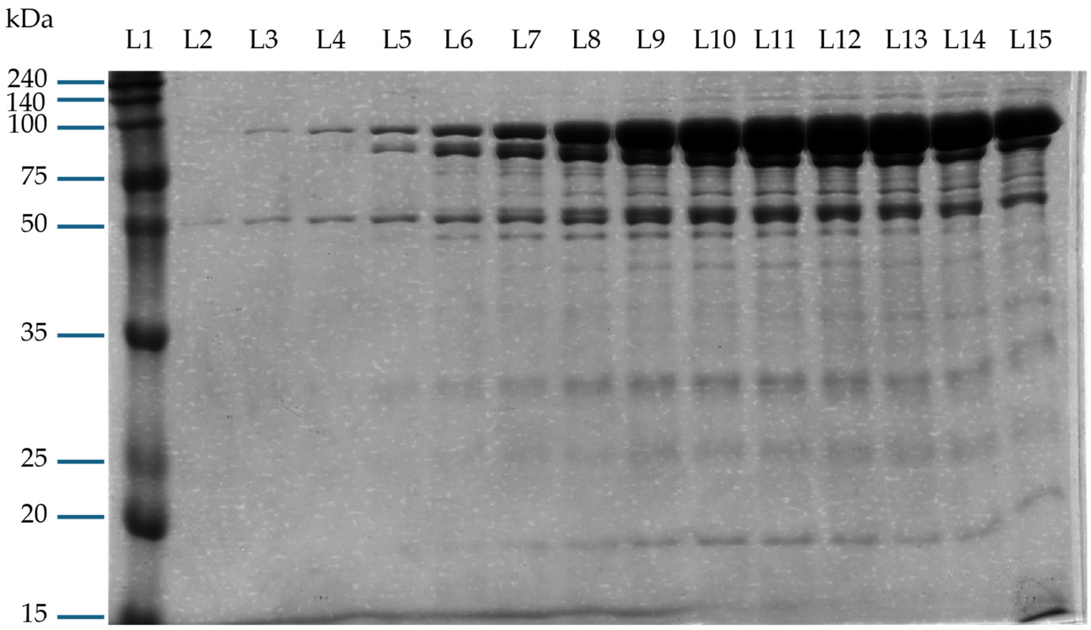


(**a**)

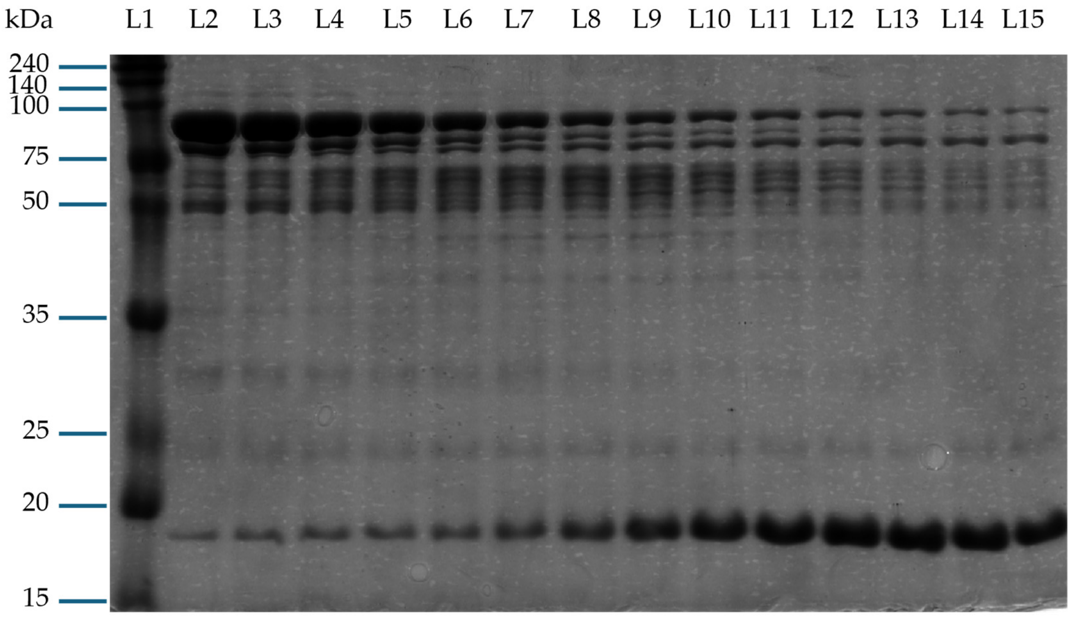


(**b**)

**Figure S1.** SDS-PAGE gel of the fractions after the first DEAE, (**a**) L1: marker, L4: sample from fraction corresponding to V = 80-82.5 mL, L12: sample from fraction corresponding to V = 100-102.5 mL, (**b**) L1: marker, L3: sample from fraction corresponding to V = 110-112.5 mL, L8: sample from fraction corresponding to V = 112.5-115 mL.

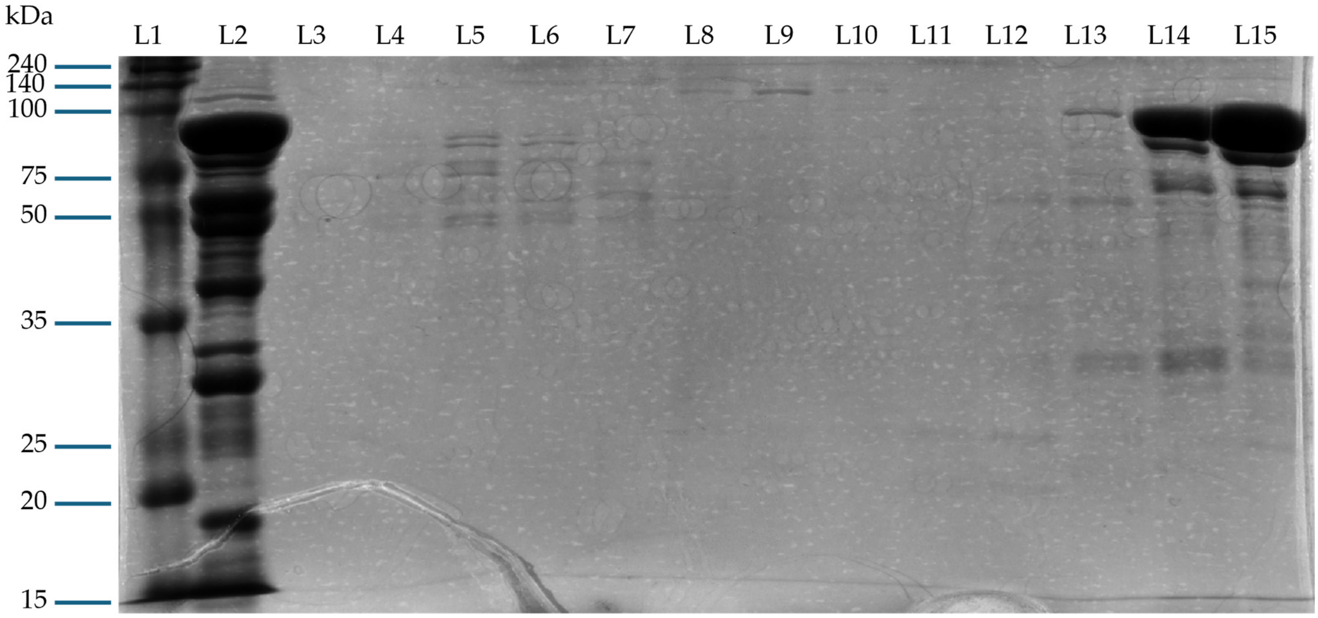


(**a**)

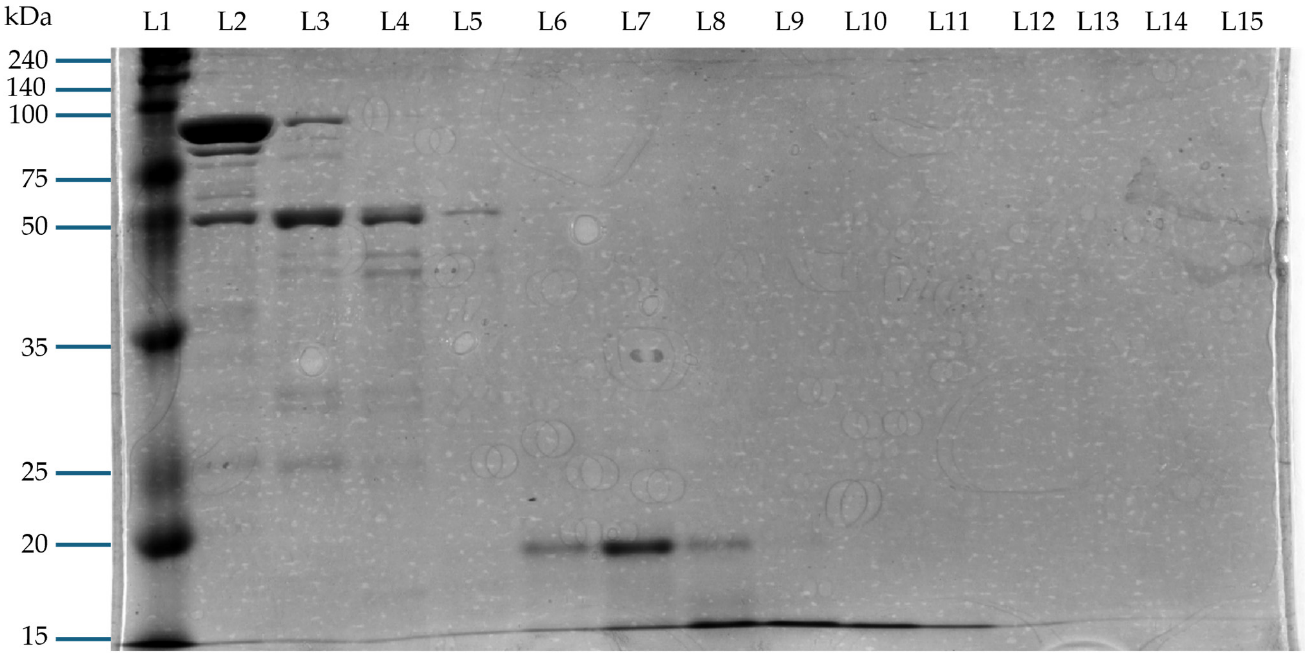


(**b**)

**Figure S2.** SDS-PAGE gel of the fractions after the SEC, (**a**) L1: marker, L14: sample from fraction corresponding to V = 72.5-75 mL, L15: sample from fraction corresponding to V = 75-77.5 mL, (**b**) L1: marker, L2: sample from fraction corresponding to V = 77.5-80 mL.

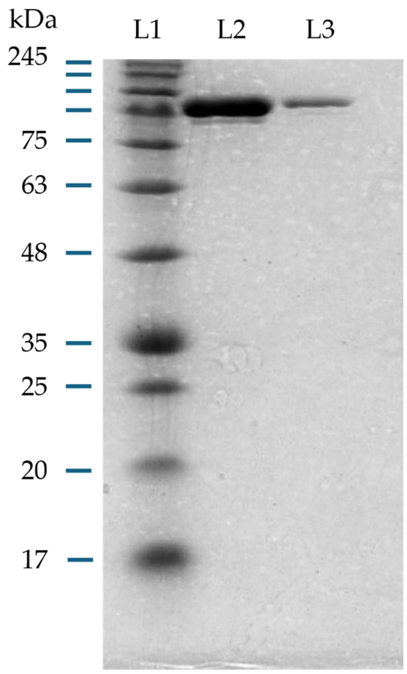


**Figure S3.** SDS-PAGE gel of the purified sample, L1: marker, L2: sLOX1 0.5 mg/mL in sodium borate 0.1M, pH 9.0.

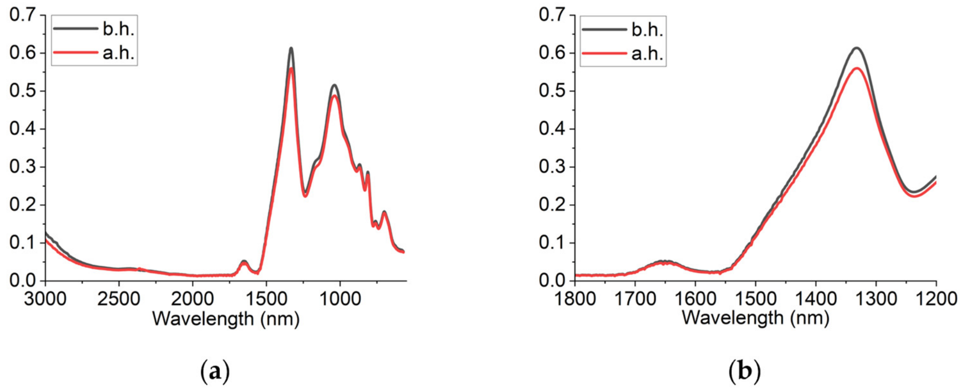


**Figure S4.** FTIR absorbance spectra of sLOX1 in 0.2 M sodium borate buffer, pH 9.0, before (b.h.) and after heating (a.h.) for wavenumber region (**a**) from 3000-500 $cm^{-1}$ and (**b**) from 1800-1225 $cm^{-1}$.

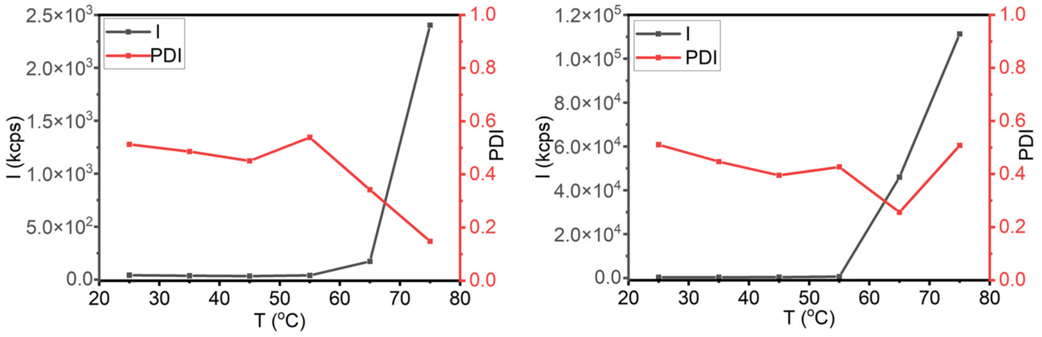


(**a**) (**b**)

**Figure S5.** DLS scattering intensity *(I)* and PDI as a function of temperature for (**a**) 0.1 mg/mL and (**b**) 1 mg/mL sLOX1 in 0.02 M sodium phosphate buffer, pH 6.8.

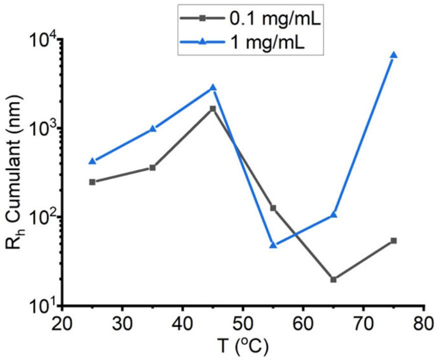


**Figure S6.** $R_h$ Cumulant as a function of temperature for 0.1 mg/mL (black) and 1 mg/mL (blue) sLOX1 in 0.02 M sodium phosphate buffer, pH 6.8.

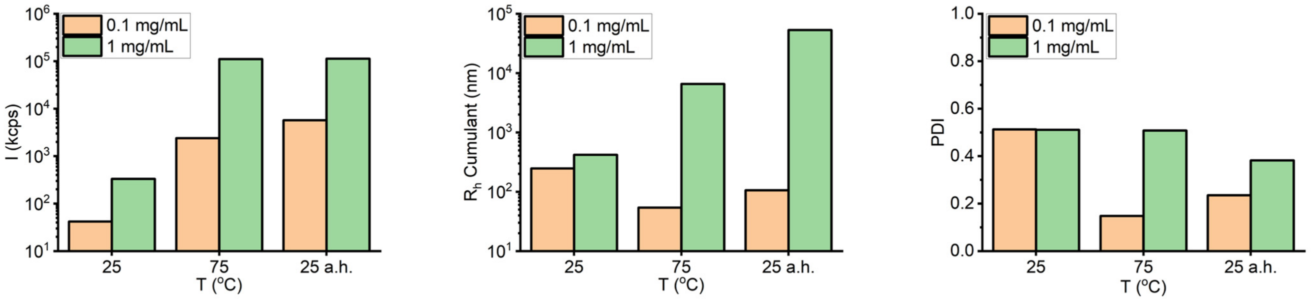


(**a**) (**b**) (**c**)

**Figure S7.** (**a**) DLS scattering intensity *(I)*, (**b**) $R_h$ Cumulant, and (**c**) PDI at room temperature (25 °C), at the highest temperature investigated (75 °C), and at room temperature after heating (25 °C a.h.) for sLOX1 0.1 mg/mL (orange) and 1 mg/mL (green) in 0.02 M sodium phosphate buffer, pH 6.8.

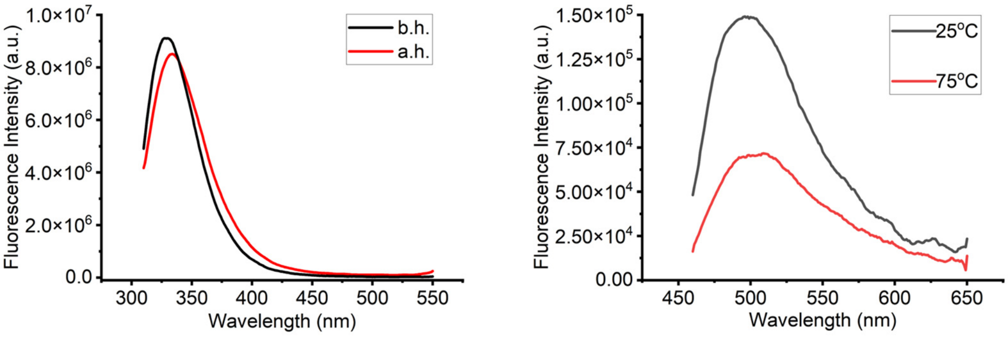


(**a**) (**b**)

**Figure S8.** (**a**) Tryptophan fluorescence spectra of a 0.1 mg/mL sLOX1 in 0.02 M sodium phosphate buffer, pH 6.8, before (b.h.) and after heating (a.h.), and (**b**) Thioflavin T (ThT) fluorescence spectra in the presence of 0.1 mg/mL sLOX1 in 0.02 M sodium phosphate buffer, pH 6.8, at 25 and 75 °C.

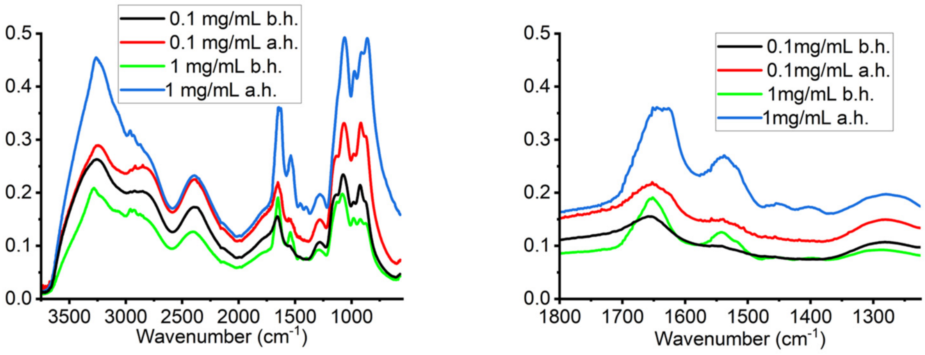


(**a**) (**b**)

**Figure S9.** FTIR absorbance spectra of sLOX1 in 0.02 M sodium phosphate buffer, pH 6.8, in two different concentrations, 0.1 and 1 mg/mL, before (b.h.) and after heating (a.h.) in the wavenumber region (**a**) 4000-500 $cm^{-1}$ and (**b**) 1800-1225 $cm^{-1}$.

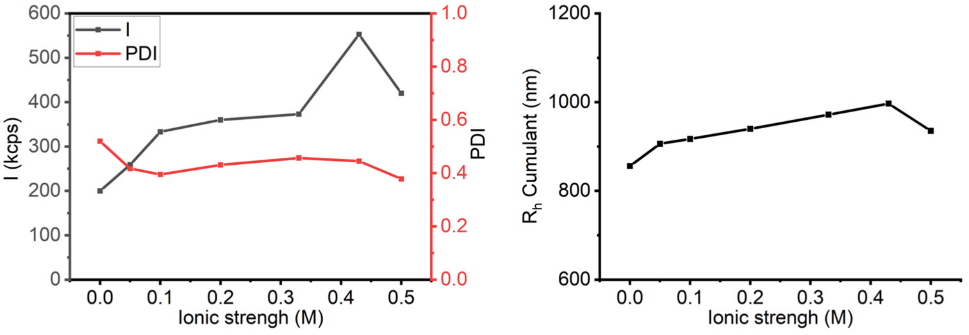


(**a**) (**b**)

**Figure S10.** (**a**) DLS scattering intensity *(I)* and PDI, and (**b**) $R_h$ Cumulant values as a function of ionic strength (NaCl) for sLOX1 0.1 mg/mL in 0.02 M sodium phosphate buffer, pH 6.8.

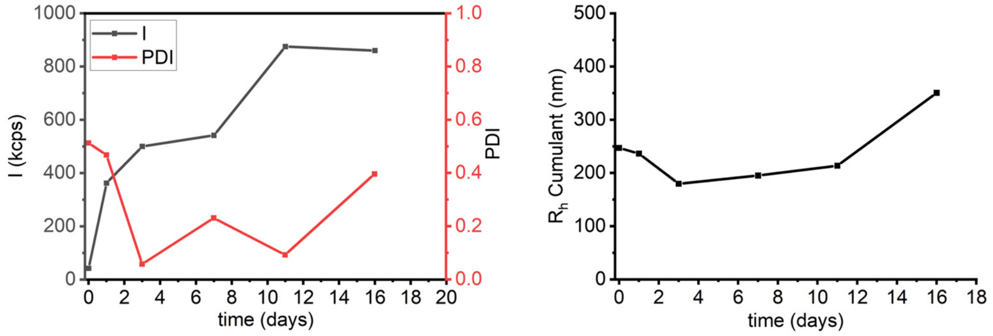


(**a**) (**b**)

**Figure S11.** (**a**) DLS scattering intensity *(I)* and PDI, and (**b**) $R_h$ Cumulant as a function of time (days) for sLOX1 0.1 mg/mL in 0.02 M sodium phosphate buffer, pH 6.8.

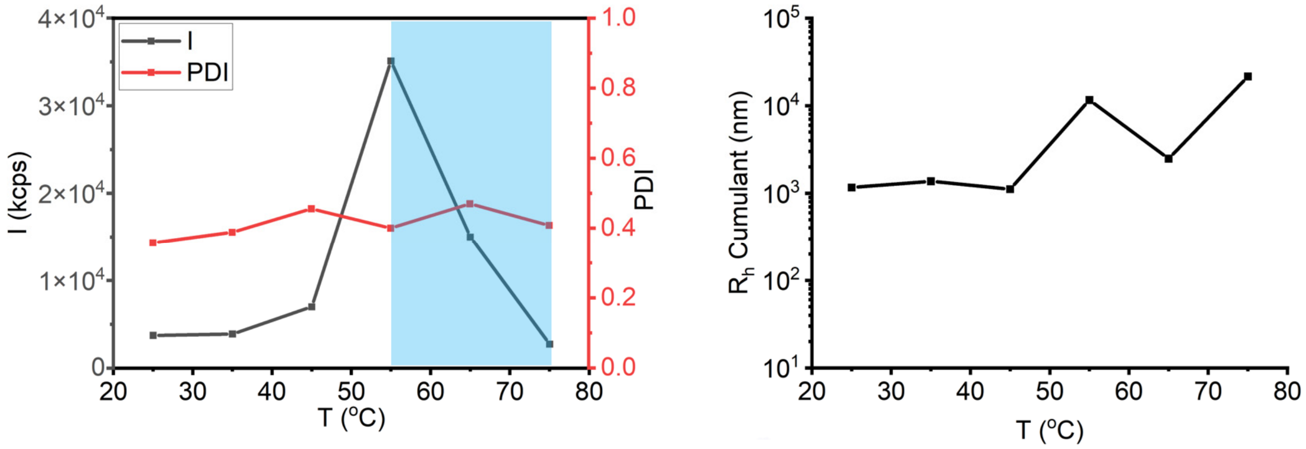


(**a**) (**b**)

**Figure S12.** (**a**) DLS scattering intensity *(I)* and PDI, and (**b**) the $R_h$ Cumulant as a function of temperature for sLOX1 0.1 mg/mL in 0.2 M sodium acetate buffer, pH 4.6. The shaded region indicates the temperature range at which the sample became opaque.

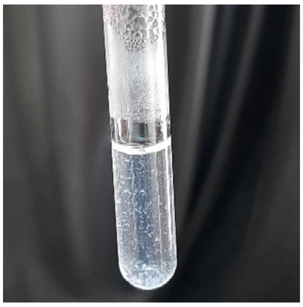

**Figure S13.** Precipitates present in 0.1 mg/mL sLOX1 solution in 0.2 M acetate buffer, pH 4.6, at 55 °C.

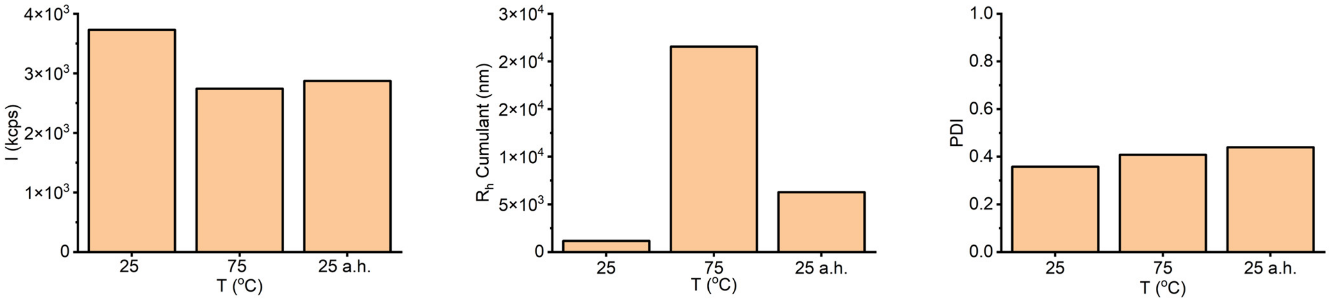


(**a**) (**b**) (**c**)

**Figure S14.** (**a**) DLS scattering intensity *(I)*, (**b**) $R_h$ Cumulant, and (**c**) PDI at room temperature (25 °C), at the highest temperature investigated (75 °C), and at room temperature after heating (25 °C a.h.) for sLOX1 0.1 mg/mL in 0.2 M sodium acetate buffer, pH 4.6.

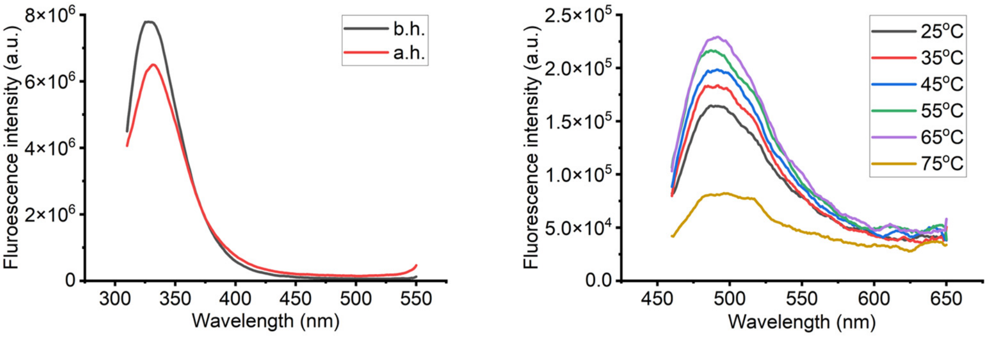


(**a**) (**b**)

**Figure S15.** (**a**) Tryptophan fluorescence spectra of a 0.1 mg/mL sLOX1 in 0.2 M sodium acetate buffer, pH 4.6, before (b.h.) and after heating (a.h.), and (**b**) Thioflavin T (ThT) fluorescence spectra in the presence of 0.1 mg/mL sLOX1 in 0.2 M sodium acetate buffer, pH 4.6, during heating.

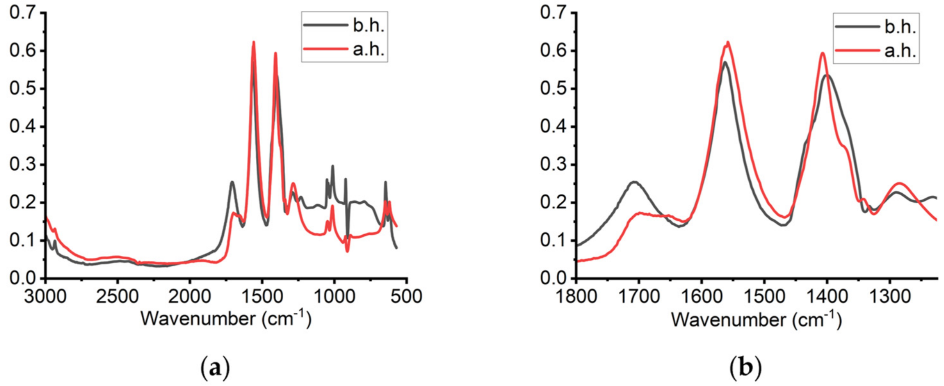


**Figure S16.** FTIR absorbance spectra of sLOX1 in 0.2 M sodium acetate buffer, pH 4.6, before (b.h.) and after heating (a.h.) in the wavenumber region (**a**) 3000-500 $cm^{-1}$ and (**b**) 1800-1225 $cm^{-1}$.

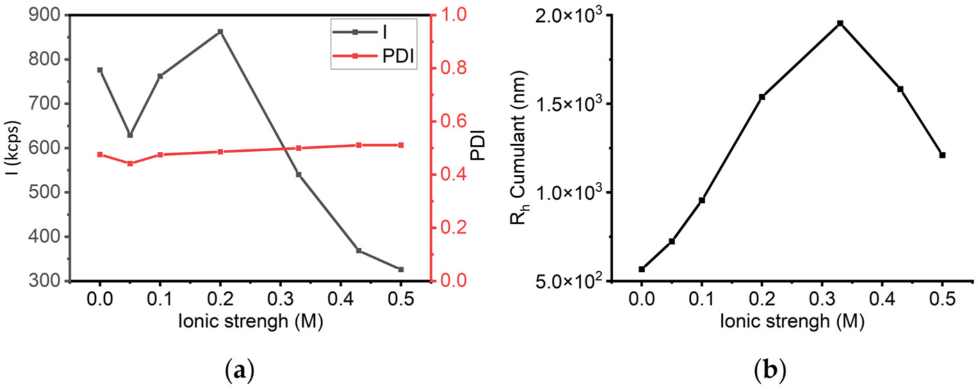


**Figure S17.** (**a**) DLS scattering intensity *(I)* and PDI, and (**b**) the $R_h$ Cumulant as a function of ionic strength (NaCl) for sLOX1 0.1 mg/mL in 0.2 M sodium acetate buffer, pH 4.6.

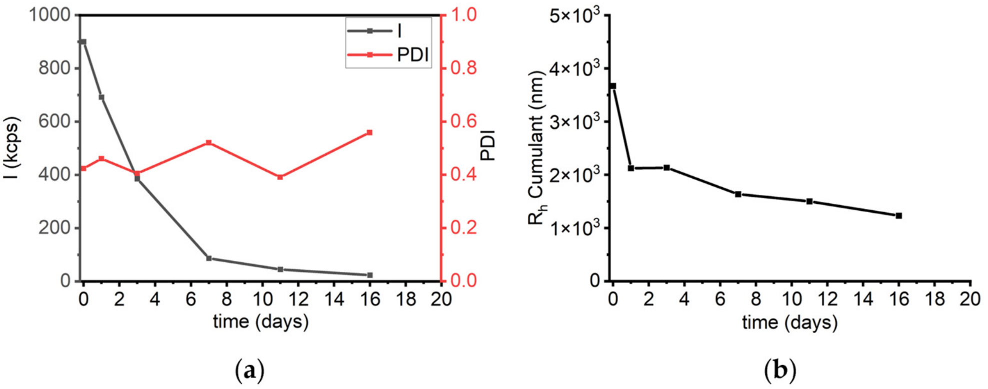


**Figure S18.** (**a**) DLS scattering intensity *(I)* and PDI, and (**b**) the $R_h$ Contin as a function of time (days) for sLOX1 0.1 mg/mL in 0.2 M sodium acetate buffer, pH 4.6.

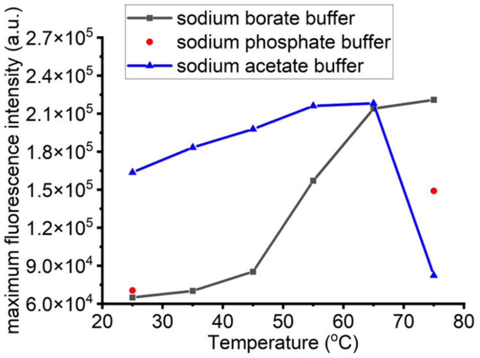


**Figure S19**. Maximum fluorescence intensity as a function of temperature in the presence of ThT for sLOX1 0.1 mg/mL in 0.2 M sodium borate buffer, pH 9.0 (black line), in 0.02 M sodium phosphate buffer, pH 6.8 (red line), and in 0.2 M sodium acetate buffer, pH 4.6 (blue line).
